# Knee Fat Pad Volumes in Patients with Hemophilia and Their Relationship with Osteoarthritis

**DOI:** 10.1155/2017/1578623

**Published:** 2017-12-05

**Authors:** Annette von Drygalski, Katherine C. Rappazzo, Richard F. W. Barnes, Eric Y. Chang

**Affiliations:** ^1^Division of Hematology/Oncology, Department of Medicine, University of California San Diego, San Diego, CA, USA; ^2^Department of Molecular and Experimental Medicine, The Scripps Research Institute, La Jolla, CA, USA; ^3^School of Medicine, University of California San Diego, San Diego, CA, USA; ^4^Radiology Service, VA San Diego Healthcare System, San Diego, CA, USA; ^5^Department of Radiology, University of California San Diego, San Diego, CA, USA

## Abstract

Hemophilic arthropathy is a progressive, disabling condition with poorly understood pathobiology. Since there is an emerging interest to study the role of intra-articular fat pad size and biology in arthritic conditions, we explored fat pad volume changes in hemophilic arthropathy and to what extent they differed from osteoarthritis. We matched a cohort of 13 adult patients with hemophilic arthropathy of the knee with age- and gender-matched cohorts without osteoarthritis (“control cohort”) and with the same degree of radiographic osteoarthritis (“OA cohort”) in 1 : 2 fashion. Infrapatellar fat pad (IPFP) and suprapatellar fat pad (SPFP) volumes were calculated based on magnetic resonance imaging and differences in fat pad volumes, demographics, height, weight, and osteoarthritis scores were evaluated. Fat pad volumes were positively associated with body size parameters in all three cohorts but were unaffected by the degree of osteoarthritis. While IPFP volumes did not differ between cohorts, SPFP volumes expanded disproportionally with weight in hemophilia patients. Our observations indicate that IPFPs and SPFPs behave biologically differently in response to different arthritic stimuli. The exaggerated expansion of the SPFP in hemophilia patients highlights the importance of further studying the implications of fat pad biology for progression of hemophilic arthropathy.

## 1. Introduction

**Figure 1:**
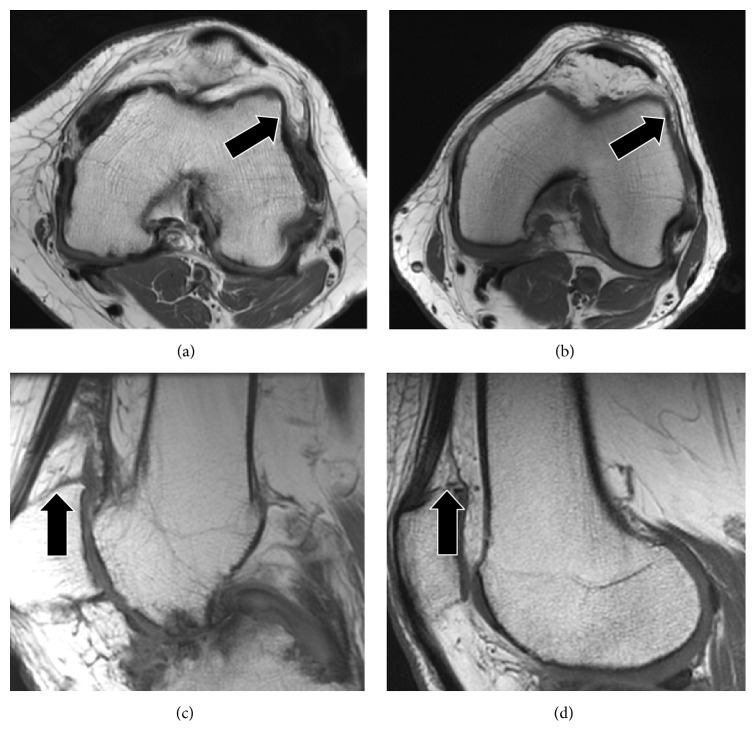
T1-weighted MR images showing different infrapatellar fat pad (IPFP) and suprapatellar fat pad (SPFP) appearances. (a) 48-year-old man with severe hemophilia B demonstrates an enlarged IPFP with prominent extension into the distended lateral patellofemoral recess (arrow). (b) 70-year-old man with mild hemophilia A shows a more regular appearance of the IPFP with only mild extension into the lateral patellofemoral recess (arrow). (c) 38-year-old man with severe hemophilia A demonstrates an enlarged and irregular SPFP (arrow). (d) 67-year-old man with mild hemophilia A shows a more regular appearance of the SPFP (arrow).

Hemophilia is an X-linked bleeding disorder due to a partial or complete deficiency of clotting factor VIII or IX. A major complication is the development of hemophilic arthropathy due to frequent joint bleeding, and the knee is the most commonly involved joint [1]. Hemophilic arthropathy is characterized by joint effusions, synovial inflammation, soft tissue hypertrophy, cartilage destruction, subchondral bone irregularities, and changes in vascularity caused by recurrent hemarthroses [2–4]. Mechanisms that contribute to the progression of hemophilic arthropathy are incompletely understood. Using noninvasive imaging modalities such as musculoskeletal ultrasound (MSKUS) and magnetic resonance (MR) imaging to identify and further characterize these pathologies in the routine clinical and research settings, we began to observe occasional pronounced alterations of fat pads in some hemophilic patients, in addition to the previously described abnormalities. Specifically, at times fat pads in the knees extended into the medial and lateral recesses (Figure 1). These are potential spaces, but in pathologic settings may become occupied by fluid, hypertrophied synovial structures, or intra-articular bodies. Since fat pad extension into these spaces has not been previously described to the best of our knowledge, we became interested to study to what extent fat pad volume changes occur in hemophilic joints. We were particularly interested in exploring whether fat pad alterations play a role in the pathobiology of hemophilic arthropathy. This line of investigation seemed pertinent because of a new movement to elucidate the biological role of fat pads in knee osteoarthritis (OA), postulating functions of fat pads beyond mere passive space occupiers or mechanical friction protectors [5, 6]. Towards that end, fat pads have been increasingly recognized to harbor inflammatory cells, thereby partaking in inflammatory and tissue repair processes [5, 6], as well as being a rich source of adipose-derived mesenchymal stem cells [7].

There are multiple distinct fat pads in the knee, which are all intracapsular, extrasynovial structures, but the most well studied one is the infrapatellar fat pad (IPFP; also known as Hoffa's fat pad) [8]. The IPFP is composed of fibrous and adipose tissue and is histologically more similar to visceral rather than subcutaneous fat [9]. The IPFP also behaves differently from subcutaneous fat, for example, preserving its volume in the setting of extreme starvation (whereas subcutaneous fat is eliminated) [9, 10] and maintaining adipocyte size in obesity (whereas subcutaneous adipose size increases) [11]. The IPFP has been shown to be a rich source of mesenchymal stem cells, cytokines, and adipokines, suggesting an important role in the homeostasis of the intra-articular milieu, in inflammatory processes, pain, and impaired joint function [9, 12]. Though less well studied, similar functions are attributed to other intra-articular fat pads, including the suprapatellar fat pad (SPFP; also known as the quadriceps fat pad) [5]. A relationship between adipokine/cytokine quantity and fat pad mass may exist, and, as a result, fat pad size has been investigated [9, 13, 14]. To date, there is no unifying consensus as to fat pad size in relation to protection against osteoarthritic changes and/or pain. Several studies have suggested that larger IPFP sizes may be protective against knee OA and/or pain [15–18], while other studies have found the opposite [19], or ascribed fat pad enlargement to advancing age in the setting of OA [20]. Findings on SPFP size and the relationship with OA have also been equivocal, with one study finding a positive association with femorotibial OA [21] and others finding no association with patellofemoral OA [22, 23]. Similarly, some studies evaluating SPFP size have found a positive association with pain [24, 25], while others have found no association [22, 26].

Hence, while there seems to be overall consensus that fat pad size and biology may play some biological role in the course of OA development, the degree of fat pad volume changes in OA and the biological role of such changes is not yet clear. Notably all previous studies have focused on middle-aged and elderly patients, nearly all with OA which is typically idiopathic (or primary) [27]. To the best of our knowledge, IPFP and SPFP volumes have not been characterized in secondary causes of OA, in particular, hemophilic arthropathy. The purpose of this study was to determine whether volume changes of the IPFP and SPFP are present in hemophilic arthropathy and to what extent they resemble or differ from primary OA. We reckoned that insights into fat pad biology in hemophilic arthropathy will improve our basic understanding of this condition and provide new leads for its largely obscure pathobiology. Towards this end, we determined IPFP and SPFP sizes in an adult hemophilia cohort and compared them to age- and gender-matched males without OA as well as patients matched for gender and radiographic degree of OA.

## 2. Methods

### 2.1. Patient Selection

After institutional review board approval, a retrospective search was performed on our clinical imaging database from March 2010 through February 2016 by a fellowship-trained musculoskeletal radiologist (E. Y. C., with 6 years of experience). Patients were included in this study only if they were greater than 18 years of age at the time of their exams and had both conventional radiographic (including frontal, lateral, and Merchant views) and knee magnetic resonance (MR) imaging exams performed. To create the* hemophilia cohort*, the search was modified to include the term “hemophilia” in the report, which identified 13 patients. To create the* age- and gender-matched control cohort without OA (“control cohort”)*, the ages of the patients in the hemophilia cohort were recorded and an age-matched search of all male cases was performed. Radiographic exams were sequentially reviewed, beginning with the most recent, and 26 patients were identified that showed no radiographic signs of osteoarthrosis on any view as previously defined by Kellgren and Lawrence (KL) [28]. To create the* gender- and OA-matched cohort (“OA cohort”)*, the radiographic exams in the hemophilia cohort were evaluated and the worst of the three knee compartments was scored for the degree of OA as previously defined by KL [28]. Specifically, KL grade 0 = normal; grade 1 = no joint space narrowing (JSN) or suspicious osteophytes; grade 2 = suspicious JSN and mild osteophytes; grade 3 = definite JSN, moderate osteophytes, and/or subchondral bone sclerosis; grade 4 = marked JSN, large osteophytes, and/or severe subchondral bone sclerosis [28]. Thereafter radiographic exams were sequentially reviewed, beginning with the most recent, and 26 male patients were identified, matched based on the presence of the worst KL grade in any of the three joint compartments. After patients in each cohort were identified, the medical records were reviewed and patient age, height, and weight were recorded. For all cohorts, exclusion criteria included previous knee surgery (as documented in the medical records or as apparent on MR imaging exams [29]) or substantial artifacts such as motion during the MR imaging procedure.

### 2.2. MR Image Acquisition

Patients underwent MR imaging on either a 1.5 T or 3 T system (GE Signa HDX, GE Medical Systems, Milwaukee, WI) with a knee coil. MRI protocols incorporated the following sequences: sagittal fast spin-echo (FSE) intermediate-weighted (TR/TE, 2500–3300 ms/30 ms; echo-train length of 8; 3-4 mm slice thickness; 0.4–0.5 mm interslice gap; 512 × 224 matrix; 14 cm field of view; and 1 signal average), sagittal FSE T1-weighted (480–500/10–15; echo-train length of 2; 3-4 mm slice thickness; 0.4–0.5 mm interslice gap; 320 × 256 matrix; 14 cm field of view; and 1 signal average), sagittal FSE T2-weighted with fat suppression (2200–4500/60–70; echo-train length of 12; 3-4 mm slice thickness; 0.4–0.5 mm interslice gap; 320 × 224 matrix; 14 cm field of view; and 1 signal average), coronal FSE T2-weighted with fat suppression (2900–5500/70, echo-train length of 8, 4-mm slice thickness, 0.4–1 mm interslice gap, 320 × 256 matrix, 14-cm field of view, and 1 signal average), coronal FSE T1-weighted (366–600/11–20; echo-train length of 2; 4-mm slice thickness, 0.4–1 mm interslice gap, 320 × 256 matrix, 14-cm field of view, and 1 signal average), and axial FSE intermediate-weighted with fat suppression (3250–3500/35–40; echo-train length of 8; 4-mm slice thickness; 0.4–1 mm interslice gap; 384 × 256 matrix; 15-cm field of view; and 2 signal averages).

### 2.3. MR Image Analysis

All images were analyzed using a standard clinical PACS system (Agfa IMPAX, Ridgefield Park, NJ). Initially, a one-hour training session on segmentation of the IPFP and SPFPs was performed between the fellowship-trained musculoskeletal radiologist (E. Y. C., with 6 years of experience) and a fourth-year medical student (K. C. R.) using routine knee MR imaging exams not included in the above cohorts. The training included identification of fat pad boundaries (indentations or clefts in the fat pads were not included in the segmented regions), use of the multiplanar triangulation feature to increase confidence when identifying the peripheral borders of the fat pads, and use of the “Markup Freeform” electronic measurement tool. Subsequently, the radiologist sorted all cases in alphabetical order and assigned case numbers to maintain blinding. Thereafter, the medical student manually measured and recorded the areas of the IPFP and SPFPs on multiple slices using the sagittal T1-weighted sequences. To assess reliability, the radiologist also measured and recorded the areas on the first nine cases. To calculate fat pad volumes, measured areas on individual slices were multiplied by the sum of slice thickness and interslice gap and all volumes were added together for a total volume. In addition, tibial plateau bone area was measured on axial T1-weighted MR images, as previously described [30, 31].

### 2.4. Statistical Analysis

The Shapiro-Wilk test was used to assess normality in the fat pad volumes. The Kruskal-Wallis test was used to assess differences in IPFP and SPFP volumes among the three groups. Correlations between fat pad volumes, demographics, and tibial plateau bone areas were evaluated for all patients and cohorts with Spearman correlations.

We examined the differences in each outcome between cohorts using linear regression models adjusted for each covariate. In these models the three cohorts were distinguished by the categorical or indicator variable cohort. The no-OA (control) cohort was the reference category. We first tested for interactions between cohort and each covariate. The covariates were age, height, weight, logBMI, tibial plateau bone areas, and KL grade. We examined KL as both an ordinal variable and as a binary variable (KLBinary) with OA defined as grade ≥2 [32]. If there were no interactions between cohort and any covariate then we tested for confounders by adding each covariate by itself to a model which had just cohort as an indicator variable. The covariate that caused the largest change in the values of the regression coefficients for cohort was considered the most important confounder [33].

When there was an interaction we constructed a model to describe the fat pad volume in terms of age, height, weight, tibial plateau bone area, or KL grade while retaining cohort as the indicator variable to distinguish the three cohorts.

Paired Student's *t*-tests and two-way mixed intraclass correlation (ICC) coefficients were used to assess interrater reliability. Statistical analyses were performed using SAS Version 9.4 (Cary, NC) and SPSS Version 21.0 (Chicago, IL).

## 3. Results

### 3.1. Subjects

Five patients in the cohort which was matched to the hemophilia group based on gender and radiographic degree of OA were excluded due to previous arthroscopic surgery. In total, 60 patients were included in the three cohorts (Table 1). Although a variety of indications for the knee MRIs were seen, pain was present as an indication for all patients.

### 3.2. IPFP Volumes

**Figure 2:**
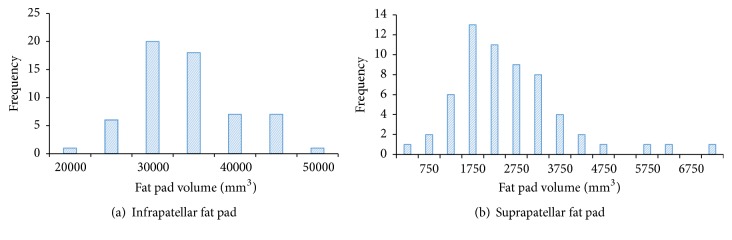
Frequency distributions for infrapatellar and suprapatellar fat pads.

IPFP volumes were normally distributed (*p* = 0.102) but slightly skewed to the right (Figure 2(a)). Using IPFP as the outcome resulted in regression models with skewed residuals that were normalized when logIPFP was used as the outcome. Therefore we used logIPFP in all regression models.

Although median IPFP volume was lower for the hemophilia cohort, there were no differences between the three cohorts (*p* = 0.704, Table 2). No association was found between IPFP volume and age, although positive correlations were shown between IPFP volumes and height, weight, BMI, and tibial plateau bone area (Supplemental Table  1, in Supplementary Material available online at https://doi.org/10.1155/2017/1578623).

**Figure 3:**
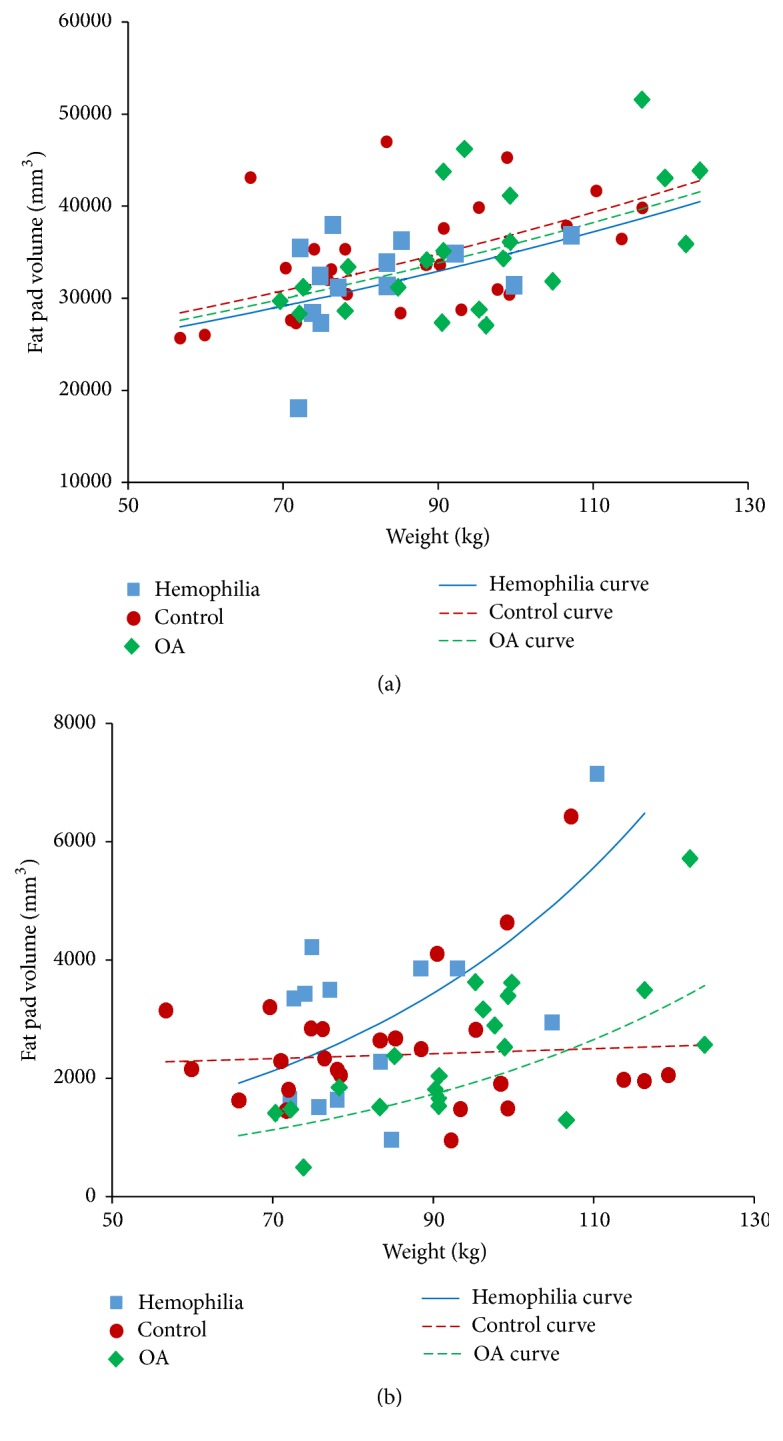
Associations between fat pad volumes and weight by cohort. (a) Infrapatellar fat pad; the lines show the regression model in Table 3. (b) Suprapatellar fat pad; the curves show the regression model in Table 4.

With logIPFP as the outcome the unadjusted regression coefficient for OA versus the control group was 0.027 (95% CI: −0.080, 0.133), while that for the Hemophilia group versus control was −0.073 (95% CI: −0.196, 0.050) (Supplemental Table  2). Weight was the confounder that had the greatest influence upon the value of regression coefficient for OA versus the control group, while tibial plateau bone area was the confounder that most influenced the value of regression coefficient for hemophilia group versus control (Supplemental Table  2). This shows that after adjustment there is no difference between the three cohorts. The model adjusted for weight is illustrated in Figure 3(a).

### 3.3. SPFP Volumes

SPFP volumes were significantly different from the normal distribution (*p* < 0.001) (Figure 2(b)). A log transformation converted this distribution into normal (*p* = 0.365). The median SPFP volume for the hemophilia group was higher than the other two cohorts, but the difference was not significant (*p* = 0.302, Table 2). SPFP volumes were not correlated with age, height, or tibial plateau bone area, but they were correlated with weight (Supplemental Table  1).

Interactions were seen between cohort and height (*p* = 0.086) and cohort and weight (*p* = 0.087). The model that best described SPFP volume in terms of weight had cohort as the indicator variable, as well as age, weight, and the interaction of cohort and weight (*r*^2^ = 0.292, *p* = 0.005) (Table 4).

Figure 3(b) indicates that SPFP volumes increased disproportionately with weight for both the OA and hemophilia cohorts, in contrast to the control group which remained unchanged with increasing weight. The hemophilia group had larger fat pad volumes than OA and also a steeper slope with weight.

### 3.4. Reliability

Assessment of the nine cases (mean 17 and 10 slices per case for IPFP and SPFP volume measurements, resp.) analyzed by both the medical student and radiologist yielded no significant differences (IPFP, *p* = 0.393; SPFP, *p* = 0.282). ICC for volume measurements was 0.92 (*p* = 0.001) for the IPFP and 0.90 (*p* = 0.002) for the SPFP volumes.

## 4. Discussion

In this study we sought to evaluate IPFP and SPFP volumes and their determinants in hemophilic arthropathy, as well as the extent to which volumes may differ from usual OA. Ultimately, we intended to explore whether arthropathic changes associated with frequent joint bleeding have effects on fat pad size that differ from usual OA, which in turn may provide pathobiological insights to be explored in more depth in future studies.

The volumes of IPFP and SPFP appeared overall quite similar between patients with hemophilia, an age- and gender-matched control group without hemophilia or OA (“control cohort”), and a gender- and OA-matched group without hemophilia (“OA cohort”). Positive correlations were present between both fat pad volumes and body size and, in particular, patient weight. Interestingly though, while the three cohorts showed similar patterns of IPFP volume increase in relation to weight, they differed with respect to SPFP and weight. SPFP volume appeared to increase much more steeply with weight in hemophilia patients compared to the OA cohort. In contrast, SPFP volume did not change at all with increasing weight in the control patients (Figure 3). Thus, the difference in frequency distributions of fat pad volumes between IPFP and SPFP is explained by SPFP volumes increasing disproportionately as weight increases in hemophilia patients, resulting in a longer tail to the right-hand end of the SPFP distribution (Figure 2).

These findings contribute to our understanding of fat pad biology on several levels. First, the fact that SPFP volume, but not IPFP volume, expanded disproportionally in relation to weight indicates that fat pads in joints are not equal and appear to react differently to certain stimuli. Second, the extent of such reactions may be influenced by the underlying arthritic condition, as exemplified by a much more pronounced SPFP expansion in relation to weight in the case of hemophilia patients compared to the OA cohort. Since both cohorts were matched for the degree of radiographic OA, other factors that are not yet understood must contribute to the volume change in hemophilic joints. Third, weight, known to be a risk factor for OA [34, 35], may not just be detrimental due to mechanical joint overload but also indirectly influence joint health through biological processes facilitated by reactive fat pad tissue.

Our finding of a positive proportional association between IPFP size and weight is consistent with those from other investigators [15, 16]. In this context some [19], but not all, studies found positive associations between IPFP size and worsening patellofemoral OA [15–18]. Therefore, it remains a current subject of debate if larger IPFP sizes are protective against OA [15–18] or not. In contradistinction to the better studied IPFP, little is known regarding SPFP volume changes in relation to weight or arthritic conditions, and, to date, SPFP volume has not been quantitatively investigated. A review of previous studies that have evaluated mass effect based on a convex posterior border yields equivocal results, with some studies finding no association with patellofemoral OA [22, 23] and others finding a positive association with worsening femorotibial OA [21]. Of note, Schwaiger et al. demonstrated an association between MR signal intensity alteration in the SPFP with degeneration of the patellofemoral joint [23], possibly related to edema and inflammation, while Tsavalas and Karantanas found no association [22].

This study is the first to evaluate both IPFP and SPFP volumes simultaneously, and our results provide evidence of a biological difference between the two intra-articular fat pads that appear at least in part influenced by the underlying arthritic condition. These findings may become meaningful in view of very recent work from Muñoz-Criado et al., who demonstrated that stem cells derived from the SPFP demonstrate higher biological activity compared with those from the IPFP with overall greater proliferative, chondrogenic, and osteogenic rates [36]. Towards that end, the initial clinical observation of unusual fat pad expansion (Figure 1) appears to be mostly due to expansion of the SPFP, not so much the IPFP. Though speculative, the observed disproportional SPFP volume expansion in hemophilia patients may be conceptually associated with higher biological reactivity caused by frequent bloody joint effusions, a feature not present in usual OA. Communication with the joint recesses usually results in fluid accumulation in the suprapatellar bursa directly abutting the SPFP, whereas there is little or no communication with areas surrounding the IPFP. Clearly, future studies are necessary to determine the biological meaning of SPFP expansion in hemophilia and potential protective or detrimental effects on joint health in this patient population in order to advance treatment options accordingly.

Past research efforts have been geared more towards the exploration of biology, mechanics, and volume changes of the IPFP rather than SPFP, possibly due to its involvement in several orthopedic conditions like impingement syndrome. The observations presented here shine light on the fact that the SPFP may also play an important role in the pathobiology of arthritic conditions that warrants further investigation. Of interest, the SPFP has also recently been implicated in a clinical impingement syndrome [37], and it will be also important to address prevalence and symptoms in the hemophilic population in future studies, in addition to molecular biological investigations.

There are limitations to our study. First, our study cohort size was small, resulting in reduced error degrees of freedom in the multivariable models. Each of the final models explained less than a third of the variation in fat pad volumes. However, we are a tertiary/quaternary referral center for patients with hemophilia and we included all patients that could be identified in our electronic clinical imaging database, which was catalogued beginning in 2010. In addition, we attempted to double the sizes of the other cohorts to increase the study size. Second, our study included only male subjects to eliminate gender effects, since hemophilia is very rare in women. Our findings may therefore not be applicable broadly to the female population with primary OA. This is important since it has been reported previously that fat pad size may be influenced independently by gender [16, 18]. Finally, images were acquired on either a 1.5 T or a 3 T MRI system and differences between image parameters may influence the results.

In conclusion, IPFP and SPFP volumes are positively associated with weight. IPFP volumes did not differ between hemophilia and control or OA-matched cohorts, suggesting that neither recurrent hemarthroses nor the presence of OA affects IPFP volumes. In contrast, SPFP volumes appear to differ between cohorts in relation to weight and the underlying arthritic condition, with a disproportional volume expansion in hemophilic joint disease. Notwithstanding unknown biological significance, our results support the concept that the IPFP and SPFP are not equivalent and that SPFP volume expansion kinetics differ between arthritic conditions. Future studies should therefore evaluate these two biologically relevant entities in tandem.

## Supplementary Material

Supplemental Table 1. Spearman rank correlations show associations between each type of fat pad and age and body dimensions. Infrapatellar fat pad volumes were positively associated with height, weight, BMI and tibial plateau bone area. In contrast, suprapatellar fat pad volumes were positively associated only with weight and BMI.
Supplemental Table 2. Examination of the effect of confounders upon the regression coefficients for log(IPFP) in relation to cohort. The first row of the table shows the unadjusted coefficients which indicate no difference between OA vs control, and none between hemophilia vs control. Subsequent rows show the coefficients adjusted for each covariate. The covariate that had the greatest effect upon the regression coefficient is shown in bold. Weight was the confounder with the greatest effect for OA vs control, while tibial bone plateau bone area had the greatest effect on the coefficient for hemophilia vs control. The coefficients adjusted for weight and tibial bone plateau bone area show that there is no difference between the cohorts.

## Figures and Tables

**Table 1:** Demographics of the three cohorts.

Cohort	*N*	Mean age ± SD (years)	Mean height ± SD (meters)	Mean weight ± SD (kilograms)
Hemophilia	13	41.8 ± 14.7	1.8 ± 0.1	82.5 ± 11.1
Matched to cohort 1 based on age and gender, but no OA	26	42.1 ± 14.5	1.8 ± 0.1	85.9 ± 16.4
Matched to group 1 based on gender and OA	21	62.3 ± 10.1	1.8 ± 0.1	94.5 ± 16.1

OA, osteoarthrosis; SD, standard deviation.

**Table 2:** Infrapatellar and suprapatellar fat pad volumes for each cohort.

Cohort	Median IPFP volume, IQR (mm^3^)	Median SPFP volume, IQR (mm^3^)
Hemophilia	3,2450	3,356
	31,162–35,476	1,649–3,859
Matched to cohort 1 based on age and gender, but no OA	33,449	2,221
	30,419–37,826	1,908–2,836
Matched to group 1 based on gender and OA	34,120	2,128
	29,715–41, 168	1,540–3,166
*p*	0.704	0.302

IPFP, infrapatellar fat pad; SPFP, suprapatellar fat pad; IQR, interquartile range; OA, osteoarthrosis.

**Table 3:** Regression model for log(infrapatellar fat pad volume) on cohort and weight.

Covariates	Regression coefficient	Standard error	*p*
Intercept	9.908	0.127	<0.001
Cohort:			
Control (no OA)	0		
OA	−0.029	0.048	0.584
Hemophilia	−0.055	0.054	
Weight (kg)	0.006	0.001	<0.001
*r* ^2^	0.293		
*p*	<0.001		

IPFP, infrapatellar fat pad; kg, kilogram; OA, osteoarthrosis.

**Table 4:** Regression model for log (suprapatellar fat pad volume) on cohort, age, and weight, with an interaction term for weight*∗*cohort.

Covariates	Regression coefficient	Standard error	*p*
Intercept	7.238	0.479	<0.001
Cohort:			
Control (no OA)	0		
OA	−2.102	0.763	0.022
Hemophilia	−1.658	1.035	
Age (years)	0.008	0.004	0.065
Weight (kg)	0.002	0.005	0.001
Weight*∗*cohort:			
Control (no OA)	0		
OA	0.020	0.008	0.034
Hemophilia	0.022	0.012	
*r* ^2^	0.292		
*p*	0.005		

IPFP, infrapatellar fat pad; kg, kilogram; OA, osteoarthrosis.
