# Supplementary material for: Knee Fat Pad Volumes in Patients with Hemophilia and Their Relationship with Osteoarthritis
Source: Arthritis. 2017 Dec 5;2017:1578623. doi: 10.1155/2017/1578623 (PMC5735625; doi:10.1155/2017/1578623)
Supplement: Supplementary file 1 — Supplemental Table 1. Spearman rank correlations show associations between each type of fat pad and age and body dimensions. Infrapatellar fat pad volumes were positively associated with height, weight, BMI and tibial plateau bone area. In contrast, suprapatellar fat pad volumes were positively associated only with weight and BMI. Supplemental Table 2. Examination of the effect of confounders upon the regression coefficients for log(IPFP) in relation to cohort. The first row of the table shows the unadjusted coefficients which indicate no difference between OA vs control, and none between hemophilia vs control. Subsequent rows show the coefficients adjusted for each covariate. The covariate that had the greatest effect upon the regression coefficient is shown in bold. Weight was the confounder with the greatest effect for OA vs control, while tibial bone plateau bone area had the greatest effect on the coefficient for hemophilia vs control. The coefficients adjusted for weight and tibial bone plateau bone area show that there is no difference between the cohorts. [file 1578623.f1.pdf]

SUPPLEMENTAL TABLES

Supplemental Table 1. Spearman rank correlations between fat pad volumes and age and body dimensions.

	Infrapatellar		Suprapatellar	
Covariate	r_s	p	r_s	p
Age (years)	0.126	0.336	0.078	0.553
Height (m)	0.267	0.041	0.077	0.561
Weight (kg)	0.523	<0.001	0.277	0.034
BMI ($kg.m^{-2}$)	0.453	<0.001	0.235	0.073
Tibial plateau bone area (cm^2)	0.467	<0.001	0.099	0.452

Supplemental Table 2: Regression coefficients for logIPFP in relation to Cohort. The first row shows the unadjusted coefficient, and subsequent rows show the coefficients adjusted for each covariate. The covariate that had the greatest effect is shown in bold.

	OA vs control			Hemophilia vs control		
Covariate	Regression coefficient	95% CI	p	Regression coefficient	95% CI	p
None (unadjusted)	0.027	-0.080, 0.133	0.6171	-0.073	-0.196, 0.050	0.2413
Age (years)	-0.012	-0.141, 0.117	0.8552	-0.072	-0.195, 0.051	0.2457
Height (m)	-0.008	-0.119, 0.102	0.8794	-0.082	-0.204, 0.040	0.1830
Weight (kg)	**-0.029**	**-0.125, 0.068**	**0.5563**	-0.055	-0.164, 0.054	0.3151
logBMI ($kg.m^{-2}$)	-0.001	-0.100, 0.099	0.9844	-0.056	-0.170, 0.059	0.3326
Tibial plateau bone area (cm^2)	-0.018	-0.117, 0.081	0.7156	**-0.023**	**-0.137, 0.091**	**0.6900**
KL	0.073	-0.153, 0.300	0.5188	-0.031	-0.269, 0.207	0.7946
KLbinary	0.071	-0.100, 0.242	0.4090	-0.027	-0.213, 0.160	0.7749
